# Impact of Bayesian penalized likelihood reconstruction on quantitative and qualitative aspects for pulmonary nodule detection in digital 2-[^18^F]FDG-PET/CT

**DOI:** 10.1038/s41598-022-09904-4

**Published:** 2022-05-18

**Authors:** Niklas Lohaus, Florian Enderlin, Stephan Skawran, Alexander Maurer, Ahmad M. A. Abukwaik, Daniel Franzen, Martin W. Huellner, Michael Messerli

**Affiliations:** 1 Department of Nuclear Medicine, University Hospital Zurich, Rämistrasse 100, 8091 Zurich, Switzerland; 2 Institute of Diagnostic and Interventional Radiology, University Hospital Zurich, Zurich, Switzerland; 3 University of Zurich, Zurich, Switzerland; 4 Department of Pulmonary Medicine, University Hospital Zurich, Zurich, Switzerland; 5 Department of Internal Medicine and Pulmonary Medicine, Spital Uster, Uster, Switzerland

**Keywords:** Diagnostic markers, Oncology, Cancer imaging, Cancer screening, Lung cancer

## Abstract

To evaluate the impact of block sequential regularized expectation maximization (BSREM) reconstruction on quantitative and qualitative aspects of 2-[^18^F]FDG-avid pulmonary nodules compared to conventional ordered subset expectation maximization (OSEM) reconstruction method. Ninety-one patients with 144 2-[^18^F]FDG-avid pulmonary nodules (all ≤ 20 mm) undergoing PET/CT for oncological (re-)staging were retrospectively included. Quantitative parameters in BSREM and OSEM (including point spread function modelling) were measured, including maximum standardized uptake value (SUV_max_). Nodule conspicuity in BSREM and OSEM images was evaluated by two readers. Wilcoxon matched pairs signed-rank test was used to compare quantitative and qualitative parameters in BSREM and OSEM. Pulmonary nodule SUV_max_ was significantly higher in BSREM images compared to OSEM images [BSREM 5.4 (1.2–20.7), OSEM 3.6 (0.7–17.4); *p* = 0.0001]. In a size-based analysis, the relative increase in SUV_max_ was more pronounced in smaller nodules (≤ 7 mm) as compared to larger nodules (8–10 mm, or > 10 mm). Lesion conspicuity was higher in BSREM than in OSEM (*p* < 0.0001). BSREM reconstruction results in a significant increase in SUV_max_ and a significantly improved conspicuity of small 2-[^18^F]FDG-avid pulmonary nodules compared to OSEM reconstruction. Digital 2-[^18^F]FDG-PET/CT reading may be enhanced with BSREM as small lesion conspicuity is improved.

## Introduction

Pulmonary nodules are becoming more frequent findings in computed tomography (CT) owing to recent technical advancements of scanner technology^1^. In non-oncological patients, pulmonary nodules are usually benign (e.g., inflammatory). However, recent studies reported a malignancy rate of pulmonary nodules of up to 85% in oncological patients^2,3^. Positron emission tomography (PET)/CT using 2-deoxy-2-[^18^F]fluoro-D-glucose (2-[^18^F]FDG) has evolved as an invaluable tool for staging and therapeutic response assessment in oncological patients^4^. However, PET/CT has generally been deemed of limited value for the evaluation of small pulmonary nodules^5,6^. But since FDG-positive pulmonary nodules detected in oncological patients have a high likelihood of malignancy^7^, techniques for a reliable detection are desired.

During the last few years, digital PET/CT systems with silicon-based detector technology for improved system sensitivity have been introduced clinically^8^. Some of these systems include a novel iterative PET reconstruction algorithm, using Bayesian penalized likelihood methods (i.e., block sequential regularized expectation maximization; BSREM)^9^. The use of BSREM reportedly increases signal-to-noise ratio (SNR), signal-to-background ratio (SBR) and maximum standardized uptake value (SUV_max_) of 2-[^18^F]FDG PET lung cancer in 2-[^18^F]FDG-PET compared to other iterative reconstruction methods, including OSEM^10^. Furthermore, BSREM yielded improved subjective image quality, tumor conspicuity and image sharpness; however, a size-based analysis was not performed in this study^10^. In line with Messerli et al., Teoh et al. found that BSREM led to a significant increase in SBR and SNR compared to OSEM^11^. This increase was higher in nodules < 10 mm, alluding to potential size-based differences. Another pilot study on lung cancer staging showed that SUV_max_ was higher in lymph nodes < 10 mm when acquired on a silicon photomultiplier-based PET/CT and reconstructed with BSREM compared to images acquired on a conventional PET/CT and reconstructed with OSEM^12^. This study is contributing to the accentuated increase in PET signal in small 2-[^18^F]FDG avid-lesions with BSREM. First specific data on small pulmonary nodule BSREM reconstruction indicated an improved lesion conspicuity and an increased SUV_max_ compared to OSEM^13^. However, this was only true for BSREM reconstruction using a *β*-value of 150 (increased SUV_max_ was actually reported for *β*-values of 150 and 250). Yet, in-depth quantitative and qualitative analysis (e.g., further size-based analysis, accurate conspicuity ratings) of small pulmonary nodules in digital PET/CT are currently lacking.

Accordingly, our study aimed to evaluate (a) the quantitative impact of BSREM on SUV_max_ in 2-[^18^F]FDG-avid pulmonary nodules; and (b) whether BSREM reconstruction affects nodule conspicuity as compared to OSEM (conventional ordered subset expectation maximization) reconstruction.

## Results

Ninety-one oncologic patients were retrospectively included in our study, with a total of 144 2-[^18^F]FDG-avid pulmonary nodules. Baseline characteristics are given in Table 1. Part of the study group was shared in a previous publication on another topic^14^. There were 63 patients (69%) with one nodule, 16 (18%) with two nodules, 6 (7%) with three nodules, 2 (2%) with four nodules, 3 (3%) with five nodules, and 1 (1%) with eight nodules.

**Table 1:** Demographic data of study subjects (*n* = 91).

Female/male, *n* (%)	36 (40%)/55 (60%)
Age, years	66 ± 11 (29–87)
Body weight, kg	71 ± 17 (40–109)
Body height, m	1.70 ± 0.10 (1.48–1.94)
BMI, kg/m^2^	24.6 ± 5.1 (14.5–38.6)
Blood glucose level at time of injection, mg/dl	96 ± 21 (54–171)
Injected FDG dose, MBq	183 ± 75 (87–302)
PET/CT scan post injection time, min	62 ± 10 (45–99)
**Indication for PET/CT scan**	
Lung cancer	32 (35%)
Head and neck cancer	9 (10%)
Colon cancer	9 (10%)
Melanoma	9 (10%)
Unknown primary cancer	6 (7%)
Lymphoma	5 (5%)
Breast cancer	4 (4%)
Urogenital cancer	4 (4%)
Small bowel cancer	3 (3%)
Cholangiocarcinoma	2 (2%)
Esophageal cancer	2 (2%)
Pancreatic cancer	2 (2%)
Rectal cancer	1 (1%)
Kaposi sarcoma	1 (1%)
Soft tissue cancer	1 (1%)
Thyroid cancer	1 (1%)

Values are given as absolute numbers and percentages in parenthesis or mean ± standard deviation (range).

### Impact of BSREM on quantitative values

The results of the quantitative analysis including SUV_max_, SBR, SNR, CBR, and CNR from BSREM and OSEM datasets are given in Table 2. Pulmonary nodule SUV_max_ was significantly higher in BSREM images as compared to OSEM images (*p* = 0.0001). The same was observed for SBR, SNR, CBR, and CNR, see Table 2. For CBR and CNR, negative values were observed since nodule SUV_mean_ was smaller than the relatively constant SUV_mean_ in the descending aorta. With lower SUV_mean_ in OSEM compared to BSREM reconstruction (2.2 ± 1.85 vs. 3.5 ± 2.9, respectively), such negative values were observed more frequently in OSEM images.

**Table 2:** Results of quantitative PET image assessment, including maximum standardized uptake value (SUV_max_) of the lung nodules (*n* = 144), nodule signal-to-background ratio (SBR), nodule signal-to-noise ratio (SNR), contrast-to-background ratio (CBR), and contrast-to-noise ratio (CNR) in block sequential regularized expectation maximization (BSREM) compared to ordered subset expectation maximization (OSEM) reconstructions as reference.

	BSREM	OSEM	*p* value^a^
**SUV**_**max**_			0.0001
Mean	5.4	3.6	
Median	4.0	2.7	
Range	1.2–20.7	0.7–17.4	
**SBR**			0.0001
Mean	3.3	2.2	
Median	2.4	1.6	
Range	0.7- 15.7	0.4–8.6	
**SNR**			0.0001
Mean	21.2	14.6	
Median	15.9	10.5	
Range	3.9–89.4	2.5–75.7	
**CBR**			0.0001
Mean	1.2	0.3	
Median	0.54	− 0.06	
Range	− 0.6 to 14.7	− 0.8 to 4.5	
**CNR**			0.0001
Mean	7.1	2.1	
Median	3.0	− 0.4	
Range	− 4.9 to 67.1	− 7.2 to 37.8	

^a^Statistical analysis was performed with Wilcoxon matched pairs signed-rank test, *p* values < 0.05 were considered to be significant.

**Figure 1:**
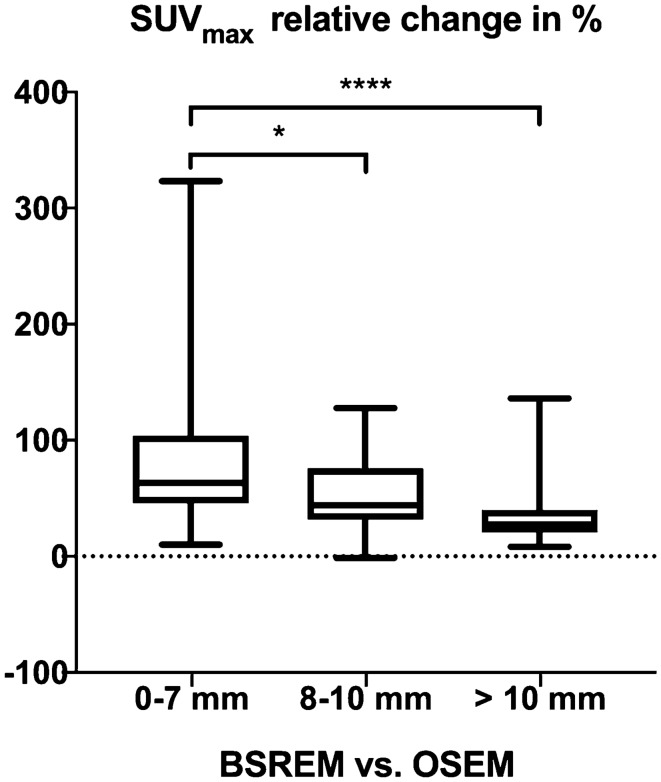
Size-based analysis of the relative increase in SUV_max_ showed a more pronounced quantitative impact in smaller nodules (≤ 7 mm) compared to larger nodules sized 8–10 mm and > 10 mm, (*p value = 0.05, ****p value = 0.0001). The whiskers of the box plot range from minimum to maximum.

**Figure 2:**
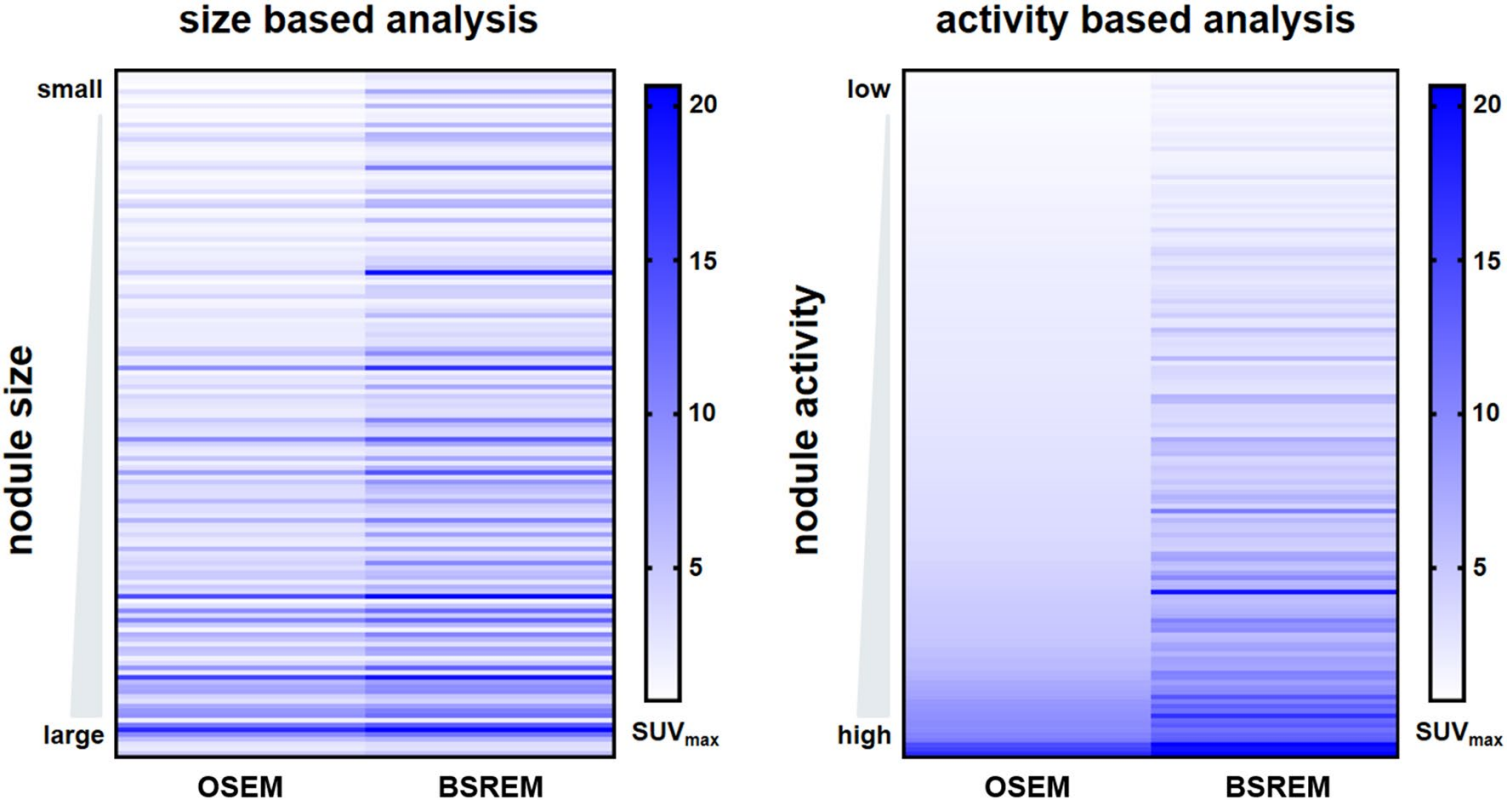
Graphical illustration of the quantitative impact of block sequential regularized expectation maximization (BSREM) reconstruction on pulmonary nodule SUV_max_ with nodules stratified by size (left) and activity (right) in ordered subset expectation maximization (OSEM). In size-based analysis, the nodule with the smallest diameter is in the top row, and the largest nodule is in the lowest row . In the activtiy-based analysis, the nodule with lowest activity in OSEM is in the top row, and the nodule with the highest activity in OSEM is in the lowest row.

In a size-based analysis, the relative increase in SUV_max_ and other quantitative parameters was more pronounced in smaller nodules (≤ 7 mm) as compared to larger ones (8–10 mm, or > 10 mm, Table 3 and Fig. 1). A graphical illustration of the quantitative impact of BSREM on SUV_max_ with pulmonary nodules stratified by size and activity is given in Fig. 2.

**Table 3:** Relative changes of maximum standardized uptake value (SUV_max_), nodule signal-to-background ratio (SBR), nodule signal-to-noise ratio (SNR), contrast-to-background ratio (CBR), and contrast-to-noise ratio (CNR) in block sequential regularized expectation maximization (BSREM) compared to ordered subset expectation maximization (OSEM) reconstructions as reference.

BSREM vs. OSEM	All nodules, *n* = 144	Nodules 1–7 mm, *n* = 46	Nodules 8–10 mm, *n* = 43	Nodules > 10 mm, *n* = 55
**Relative change**				
SUV_max_	+ 53.1 ± 44.3%	+ 79.5 ± 59.5%	+ 51.0 ± 30.7%	+ 32.5 ± 22.5%
SBR	+ 52.7 ± 45.0%	+ 78.5 ± 62.0%	+ 50.4 ± 30.4%	+ 32.9 ± 22.5%
SNR	+ 49.3 ± 42.0%	+ 72.6 ± 55.0%	+ 53.0 ± 29.4%	+ 26.8 ± 22.8%
CBR	+ 80.8 ± 626.8%	+ 169.6 ± 944.0%	+ 47.2 ± 336.4%	+ 32.8 ± 355.4%
CNR	+ 74.6 ± 587.7%	+ 153.2 ± 921.3%	+ 51.9 ± 338.9%	+ 26.5 ± 341.3%

Values are given as mean ± standard deviation.

### Qualitative values: impact of BSREM on nodule conspicuity

**Figure 3:**
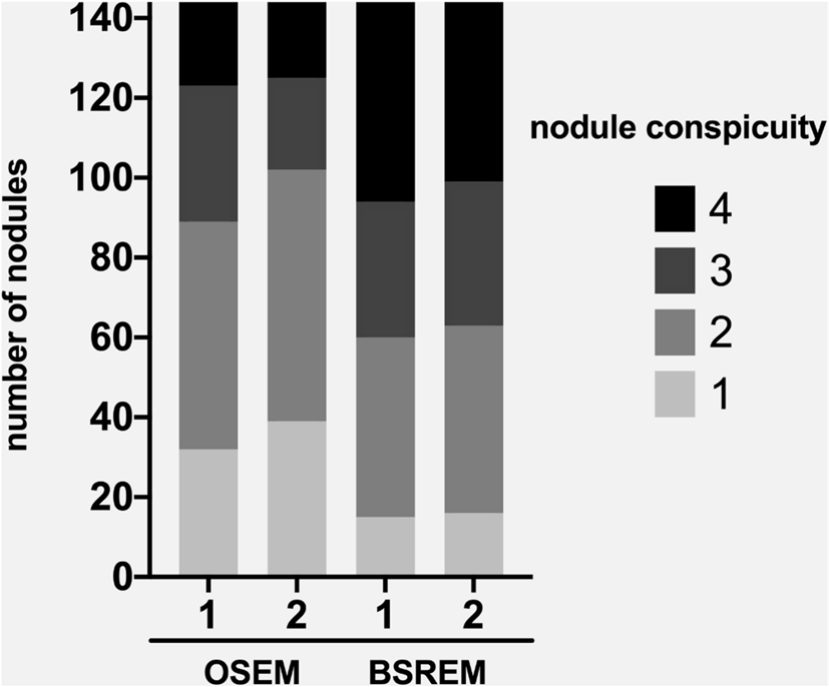
Subjective image quality ratings of reader 1 and reader 2 for ordered subset expectation maximization (OSEM) and block sequential regularized expectation maximization (BSREM) reconstruction images.

The mean conspicuity score was 2.8 for reader 1 and 2.8 for reader 2 in BSREM, which was significantly higher compared to OSEM (2.3 for reader 1 and 2.2 for reader 2; *p* both < 0.0001). Figure 3 illustrates the conspicuity score ratings for all nodules (*n* = 144) of the two readers for BSREM and OSEM reconstruction. Inter-reader agreement for nodule conspicuity with OSEM was substantial (OSEM: Cohen’s kappa = 0.747) and for BSREM almost perfect (BSREM: Cohen’s kappa = 0.846).

**Figure 4:**
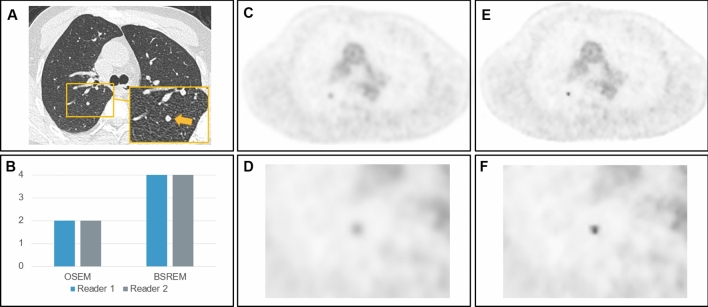
Representative images of a 63-year-old man with a body mass index of 22.2 kg/m^2^ and 75 kg body weight who underwent 2-[^18^F]FDG PET/CT for re-staging of esophageal cancer. CT images (**A**) show a newly developing 5 mm nodule in the right upper lobe. Subjective image quality ratings (**B**) of reader 1 and reader 2 for ordered subset expectation maximization (OSEM) and block sequential regularized expectation maximization (BSREM) reconstruction images indicate an increased lesion conspicuity. Axial slices at the same level showing OSEM reconstruction (**C**,**D**) and BSREM reconstruction (**E**,**F**) show the 2-[^18^F]FDG-avid nodule.

Representative images of a study subject undergoing 2-[^18^F]FDG-PET/CT for oncologic staging are given in Fig. 4.

### Clinical follow up for nodule etiology

**Figure 5:**
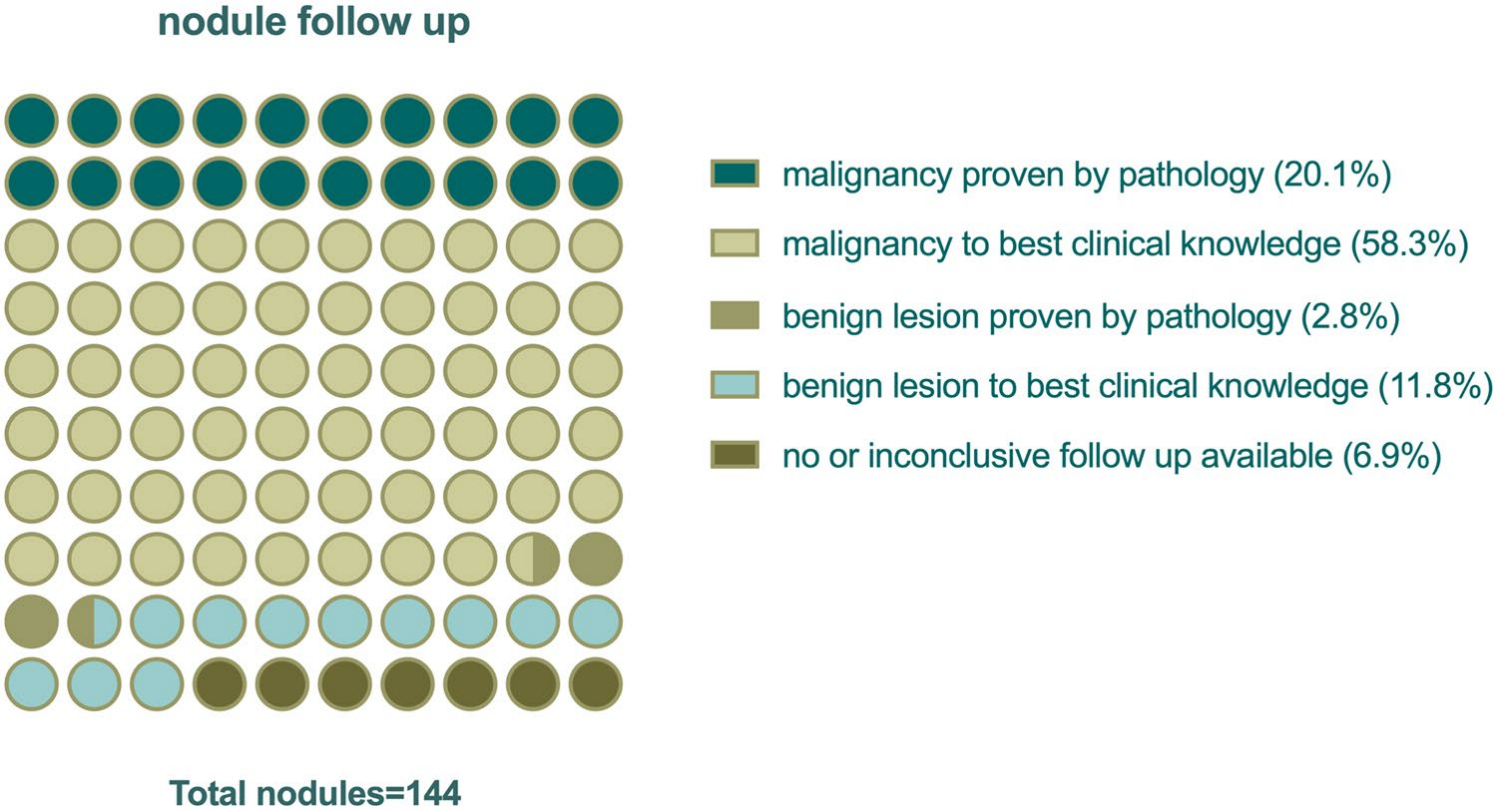
Analysis of nodule etiology during up to three years of follow-up is shown in a dot plot. The values of the parts per whole analysis are given in percent. All nodules (*n* = 144) were included.

To gain more information about the etiology of the 2-[^18^F]FDG-avid pulmonary nodules, the clinical information system of our hospital was screened for information about the etiology of the nodules, Fig. 5. Overall, 20.1% (29/144) of nodules were found to be malignant, as proven by pathology. Another 84 of 144 nodules (58.3%) were clinically suspected to be malignant (e.g., owing to growth on follow-up imaging), albeit without pathological proof. Only 2.8% (4/144) of nodules were proven by pathology to be benign. Another 11.8% (17/144) of nodules was assumedly benign, based on radiological follow-up exams. 6.9% (10/144) of nodules remained undetermined, since no or inconclusive follow-up data was available.

### Diagnostic performance based on SUV_max_ (BSREM vs. OSEM)

**Figure 6:**
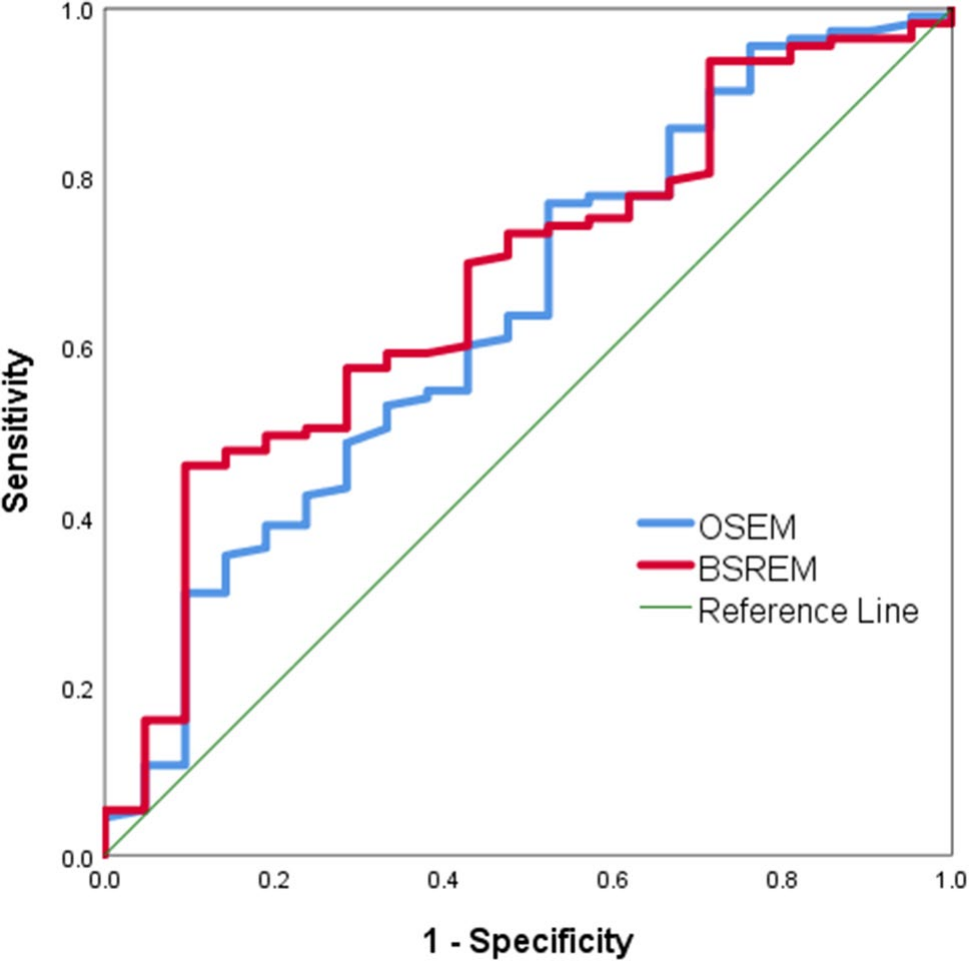
ROC curves for assesment of pulmonary nodules on OSEM and BSREM based on SUV_max_ as a single determinant of malignant etiology.

Receiver operating characteristic (ROC) curves evaluating the value of BSREM and OSEM to differentiate malignant from benign nodules based on SUV_max_ are presented in Fig. 6. The area under the curve (AUC) values were 0.639 (*p* = 0.044) and 0.675 (*p* = 0.011), respectively, with no statistically significant difference between the two algorithms (*p* = 0.128).

## Discussion

This study sought to evaluate the impact of BSREM reconstruction on the quantitative and qualitative aspects of 2-[^18^F]FDG-avid pulmonary nodules compared to conventional OSEM reconstruction on a latest-generation silicon-based digital detector PET/CT scanner.

The major findings of our study are as follows: (1) BSREM reconstruction algorithm leads to a significant increase in SUV_max_ and other quantitative parameters in small pulmonary nodules compared to OSEM, with an average increase of nodule SUV_max_ by 53%. (2) The quantitative impact of BSREM was most pronounced in the subgroup of smallest nodules (≤ 7 mm), with a mean relative increase in SUV_max_ by 80% in this subgroup. (3) BSREM yielded a higher conspicuity of pulmonary nodules than OSEM. (4) The use of BSREM did not improve the overall accuracy of 2-[^18^F]FDG PET/CT for differentiating malignant from benign nodules.

Pulmonary nodules are a frequent but unspecific CT finding in the daily routine of radiologists and nuclear medicine physicians^1^. In the Pan-Canadian Lung Cancer Screening Study (PanCan), the reported high percentage (74%) of patients with at least one pulmonary nodule was in contrast to the low percentage (5.5%) of actually malignant nodules^15^. In non-oncological subjects, the guidelines by the Fleischner Society explain how to deal with incidental pulmonary nodules on CT^16^. In oncological patients, however, there is no clear consensus, and the literature is scarce on how to manage “incidental” pulmonary nodules in these patients^17^. A recent study by Taralli et al. indicated that in oncological patients 2-[^18^F]FDG PET/CT may perform well in *ruling in* malignancy if pulmonary nodules are 2-[^18^F]FDG -avid^7^. PET/CT was also found useful for personalizing patient management by identifying the “reference” nodule deserving histological examination^7^.

For many years, 2-[^18^F]FDG PET/CT was regarded unsuitable for the assessment of small pulmonary nodules, mainly owing to the comparably low spatial resolution of PET^18^. Due to recent technological advancements, including novel digital detector systems and improved reconstruction algorithms, pulmonary nodules are now detected more frequently on PET . Indeed, in our cohort 89/144 (62%) of FDG-avid nodules were ≤ 10 mm, and 46/144 (32%) were even < 8 mm. In addition to the novel detector system, BSREM further enhances the SNR, SBR, CNR, CBR and SUV_max_, particularly of small nodules. In our cohort, the average SUV_max_ increased from 3.6 with OSEM to 5.4 with BSREM, which represents an increase by 53%. In a previous study by Teoh et al. using a photomultiplier tube PET system, it was also reported that BSREM increases the SBR/SNR as compared to OSEM in small pulmonary nodules^11^. Similar to Teoh et al., in our study the increase in SUV_max_ of pulmonary nodules in BSREM did not translate into significant differences of ROC curves using SUV_max_ as a single determinant of malignancy. In another study small (< 10 mm) suspected lymph node metastasis had higher SUV_max_ when reconstructed with BSREM compared to OSEM^12^. As a limiting factor of this finding by Economou et al.^12^ it needs to be pointed out that they did not only use two different reconstruction algorithms, but also different PET-scanners. The retrospective pilot study by Howard et al. found—besides increased SUV_max_ as a quantitative measure—also increased visual lesion conspicuity (as a qualitative measure) in 32 analyzed nodules that were previously described as “too small to characterize”^13^. Today, the study by Howard et al. is the only hint that sole “quantitative improvement” would also affect lesion conspicuity. Furthermore, it is clear that augmented quantitative accuracy in PET may not consequently translate into an improvement of clinical reading. Therefore, performance assessments of readers were included in our study to complement the quantitative approach and further validate improved conspicuity of small nodules on BSREM. We could show that PET reading may be enhanced with BSREM, since small lung lesion conspicuity was improved in our study. The improved conspicuity on BSREM may be related to the fact that the increase in SUV_max_ and the other quantitative parameters (SBR, SNR, CBR and CNR) translate into a better lesion recognition by the human eye. For the quantitative data it was previously described that BSREM increased quantitation accuracy compared to OSEM, especially in cold background regions, such as lungs^19,20^. Similarly to Teoh et al., we believe that quantitative increases in SUVmax are due to almost full convergence of BSREM, compared to the only partial convergence of OSEM (in our study two iterations were used)^9,11^. Due to the limited convergence of OSEM, the true SUV_max_ is consistently underestimated. The underestimation in OSEM is particularly pronounced in small lesions^21^. However, this underestimation can normally be mitigated if point spread function modeling is used^22^. Interestingly, we were still able to measure differences, although both OSEM and BSREM used point spread function modeling. Moreover, as described in previous phantom studies, BSREM improved the quantification accuracy especially for smaller (i.e., sub-centimeter) nodules^23^.

Our study has some limitations. First, our study group is relatively small and limited to a single center. Second, we included patients with 2-[^18^F]FDG-avid pulmonary nodules without further proof of the etiology of these nodules at the time of inclusion (i.e., malignant vs. benign). However, we feel that in an oncological cohort, any 2-[^18^F]FDG-positive nodule is potentially relevant and warrants at least follow-up imaging, considering the generally high pre-test probability of malignancy. The relevance of the 2-[^18^F]FDG-positive nodules was confirmed by our analysis of nodule etiology, since the majority (78.4%) of nodules was either pathology-proven (20.1%) or clinically highly suspected (58.4%) to be malignant (20.1%). Third, we did not reconstruct images with different *β*-values of BSREM or with different OSEM settings, which may differently affect quantitative or qualitative features of lesions in different subjects, depending for example on the individual 2-[^18^F]FDG dosage or BMI. It is expected that further iterations using OSEM and PSF may alter quantitative aspects of pulmonary nodules. However, it is well known that such high-iteration OSEM images are deteriorated by noise and are unusable for clinical PET reading.

In conclusion, BSREM results in a significant increase of SUV_max_ and improved signal-to-noise ratio in small 2-[^18^F]FDG-avid pulmonary nodules compared to conventional OSEM reconstruction. The conspicuity of small pulmonary lesions on digital detector 2-[^18^F]FDG PET/CT may be enhanced using BSREM reconstruction.

## Materials and methods

### Study subjects

Between December 2017 and March 2019, all patients included in the our study underwent a clinically indicated 2-[^18^F]FDG-PET/CT for oncological (re-)staging. We retrospectively included patients with one to ten small 2-[^18^F]FDG-avid pulmonary nodules (i.e., ≤ 20 mm size on CT). Written informed consent for the scientific use of medical data was obtained from all subjects. The local ethics committee (Kantonale Ethikkommission, Zurich, Switzerland) approved the study. The study was conducted in compliance with the International Council for Harmonisation of Technical Requirements for Pharmaceuticals for Human Use of Good Clinical Practice rules and the Declaration of Helsinki.

### PET image acquisition and reconstruction

PET/CT scans were performed using a latest generation 5-ring digital detector PET/CT scanner (GE Discovery Molecular Insights—DMI, GE Healthcare, Waukesha, WI) and a standardized clinical protocol. A body mass index (BMI)-adapted 2-[^18^F]FDG dosage protocol developed for digital PET detectors was used, as previously described in detail^24,25^, with 2-[^18^F]FDG dosage injection ranging from 1.5 MBq per kilogram to 3.1 MBq per kilogram body weight, without exceeding a maximum of 320 MBq. Participants fasted for at least 4 h prior to the scan and blood glucose level was below 160 mg/dl at the time of 2-[^18^F]FDG injection. The targeted 2-[^18^F]FDG uptake time was 60 min. A CT scan was obtained from the vertex of the skull to the mid-thighs and used for attenuation correction as well as for anatomical localization of 2-[^18^F]FDG distribution. The CT scan was acquired using an automated dose modulation technique (range 15–100 mA) with 120 kVp. After the CT scan, PET images were acquired covering the identical anatomical region. The acquisition time for PET was 2.5 min per bed position, with 6–8 bed positions per patient (depending on patient size), with an overlap of 23% (17 slices). The PET was obtained in 3D mode and slice thickness was 2.79 mm.

Two PET dataset reconstructions were generated using (1) BSREM (Q.Clear, GE Healthcare) with a default *β*-value of 450, and (2) OSEM with two iterations, 24 subsets (i.e., 48 image updates, as recommended by the vendor) and 6.4 mm Gaussian filter with time-of-flight reconstruction and point spread function modelling (OSEM; Vue Point FX with SharpIR, GE Healthcare). All PET datasets were reconstructed with a 256 × 256 pixel matrix.

### Quantitative imaging analysis

Quantitative analyses were performed by one reader (*Blinded for Review*, with 2 years of experience in radiology/nuclear medicine NL). A standard volume of interest (VOI) was used to record the maximum standardized uptake value (SUV_max_) of each pulmonary nodule in both BSREM and OSEM datasets. Nodule diameter was measured in the long-axis on axial CT slices in lung window. Similar to Teoh et al.^11^, background SUV was recorded in the right lobe of the liver (parenchymal organ background) and within the descending aorta (blood pool background) at the level of the carina, with 4.0 cm-diameter (liver) and 1.0 cm-diameter (aorta) spherical VOIs. Only liver parenchyma appearing normal on both PET and CT was used as a reference. In both backgrounds and for both reconstructions, the mean standardized uptake value (SUV_mean_) and the standard deviation of the standardized uptake value (SUV_SD_) within the VOIs were recorded. As previously described, a signal-to-background ratio (SBR) based on these measurements was calculated for each nodule, defined as the lesions’ SUV_max_ divided by the SUV_mean_ in the descending aorta^10^. The liver SUV_SD_ served as a measure of noise. Nodule signal-to-noise ratio (SNR) was defined as the lesion’s SUV_max_ divided by the liver SUV_SD_. Furthermore, the (nodule SUV_mean_ minus the SUV_mean_ in the descending aorta) divided by the SUV_mean_ in the descending aorta defined a calculated contrast-to-background ratio (CBR)^10^. Lastly, contrast-to-noise ratio (CNR) was calculated, defined as the (nodule SUV_mean_ minus the SUV_mean_ in the descending aorta) divided by the liver SUV_SD_^10^.

### Subjective imaging analysis: Assessment of nodule conspicuity

Two readers (*Blinded for Review*, with N.L. 2 and A.M. 8 years of experience in radiology/nuclear medicine) independently assessed the conspicuity of each nodule. For the nodule conspicuity, the readers rated as follows: 1, poor conspicuity of lesion; 2, fair conspicuity, 3, good conspicuity; and 4, excellent conspicuity.

### Clinical follow-up for nodule etiology analysis

The clinical information system was screened for the best available data on the etiology of the 2-[^18^F]FDG-avid pulmonary nodules. Every available information (e.g., pathology reports, radiological follow-up scans, oncology reports) were used to determine the etiology. Nodule etiology was grouped into five categories as follows: (1) malignancy proven by pathology; (2) malignancy to best clinical knowledge; (3) benign lesion proven by pathology; (4) benign lesion to best clinical knowledge; and (5) no or inconclusive follow up available.

### Diagnostic performance of SUV_max_ (BSREM vs. OSEM)

The diagnostic performance of BSREM and OSEM to differentiate malignant from benign nodules based on SUV_max_ was assessed using an ROC curve and AUC values.

### Statistical analyses

Categorical variables are expressed as proportions, and continuous variables are presented as mean ± standard deviation or median (range), depending on the distribution of values. Wilcoxon matched pairs signed-ranks test was applied for comparison of SUV_max_, SBR, SNR, CBR, and CNR values in BSREM vs. OSEM. Furthermore, the same test was also used to compare the subjective analysis of the readers (i.e., conspicuity score per nodule). Mann–Whitney U test was performed for size-based comparisons of relative increase of SUV_max_. For the post-hoc analysis, a Bonferroni-corrected *p*-value of 0.025 (0.05/number of tested size groups) was considered to indicate statistical significance. The difference ratios of SUV_max_, SBR, SNR, CBR, and CNR values in BSREM vs. OSEM datasets were calculated using the results of OSEM reconstructions as reference as follows: (variable in BSREM reconstruction minus variable in OSEM reconstruction)*100/(variable in OSEM reconstruction). All analyses were performed with statistics software (SPSS version 26.0, IBM Corporation, Armonk, NY or GraphPad Prism version 8.3.1). A two-tailed *p*-value of < 0.05 was considered to indicate statistical significance.

## Data Availability

The datasets/images used and/or analyzed in this study are available from the corresponding author upon reasonable request.

## Abbreviations

2-[^18^F]FDG: 2-Deoxy-2-[^18^F]fluoro-D-glucose
AUC: Area under the curve
BMI: Body mass index
BSREM: Block sequential regularized expectation maximization
CBR: Contrast-to-background ratio
CNR: Contrast-to-noise ratio
CT: Computed tomography
MBq: Megabecquerel
OSEM: Ordered subset expectation maximization
PET: Positron emission tomography
ROC: Receiver operating characteristic
SBR: Signal-to-background ratio
SNR: Signal-to-noise ratio
SUV_max_: Maximum standardized uptake value
VOI: Volume of interest
